# Obesity and its implications on cerebral circulation and intracranial compliance in severe COVID‐19

**DOI:** 10.1002/osp4.534

**Published:** 2021-05-27

**Authors:** Sérgio Brasil, Alessandra Covallero Renck, Fabio Silvio Taccone, Davi Jorge Fontoura Solla, Bruno Martins Tomazini, Sâmia Yasin Wayhs, Sérgio Fonseca, Estevão Bassi, Bruno Lucena, Ricardo De Carvalho Nogueira, Wellingson Paiva, Manoel Jacobsen Teixeira, Elaine Maria Frade Costa, Luiz Marcelo Sá Malbouisson

**Affiliations:** ^1^ Universidade de São Paulo Brazil; ^2^ Universitè Libre de Bruxelles Belgium

**Keywords:** cerebral hemodynamics, COVID‐19, intracranial compliance, obesity, severe acute respiratory syndrome

## Abstract

**Objective:**

Multiple factors have been identified as causes of intracranial compliance impairment (ICCI) among patients with obesity. On the other hand, obesity has been linked with worst outcomes in COVID‐19. Thus, the hypothesis of severe acute respiratory syndrome (SARS) conducing to cerebral hemodynamic disorders (CHD) able to worsen ICCI and play an additional role on prognosis determination for COVID‐19 among obese patients becomes suitable.

**Methods:**

50 cases of SARS by COVID‐19 were evaluated, for the presence of ICCI and cerebrovascular circulatory disturbances in correspondence with whether unfavorable outcomes (death or impossibility for mechanical ventilation weaning [MVW]) within 7 days after evaluation. The objective was to observe whether obese patients (BMI ≥ 30) disclosed worse outcomes and tests results compared with lean subjects with same clinical background.

**Results:**

23 (46%) patients among 50 had obesity. ICCI was verified in 18 (78%) obese, whereas in 13 (48%) of 27 non‐obese (*p* = 0,029). CHD were not significantly different between groups, despite being high prevalent in both. 69% unfavorable outcomes were observed among obese and 44% for lean subjects (*p* = 0,075).

**Conclusion:**

In the present study, intracranial compliance impairment was significantly more observed among obese subjects and may have contributed for SARS COVID‐19 worsen prognosis.

## 1 INTRODUCTION

The association between obesity (body mass index > 30kg/m^2^) and intracranial hypertension (ICH) has been widely described.^1^, ^2^, ^3^ Elevation in the intracranial pressure (ICP) may reduce intracranial compliance (ICC), what is the equilibrium among intracranial content (brain, blood volume and cerebrospinal fluid),^4^ impacting cerebral perfusion and cellular metabolism.^5^ Several mechanisms linking obesity with chronic ICH have been proposed, mainly as disturbances of cerebrospinal fluid circulation,^6^, ^7^ dysregulation of the metabolic neuroendocrine axis,^8^ compression of thoracic and abdominal organs impairing cerebral venous return,^9^ sleep apnea leading to cerebral hemodynamics disorders (CHD)^6^, ^10^, ^11^ and brain temperature elevation.^12^ Additionally to genetic and epigenetic determinants,^13^ these factors may also play a role in increasing risks of neurodegenerative diseases (NDD) development in this population.

ICH prevalence among general population has been not widely studied to the date, especially because techniques to assess ICP require skull opening for catheter introduction, what is ethically not recommended. Nevertheless, 90–95% of patients with idiopathic intracranial hypertension (IIH) symptoms have obesity.^14^ Hence, the hypothesis of obesity coexisting with a lifetime regimen of ICH and consequently ICC impairment (ICCI) if obesity is untreated^15^ becomes suitable.

At the current COVID‐19 pandemic, obesity has been considered a prognostic risk factor, with particular monitoring and earlier respiratory support recommended for these patients.^16^, ^17^, ^18^ Considering this background explained above, for obese patients with COVID‐19 severe respiratory syndromes, the hypothesis of ICCI and CHDbeing higher when compared with lean patients is feasible. The objective of the present study was to evaluate the prevalence of ICCI and CHD in correlation with short‐term clinical outcomes as death and mechanical ventilation weaning (MVW) in severe COVID‐19 among patients with and without obesity.

## 2 METHODS

### 2.1 Study design

A single center, observational and prospective study in six intensive care units (ICUs) of Hospital das Clínicas, São Paulo University, Brazil, from May to June 2020 was conducted. All methods were performed in accordance with the relevant guidelines and regulations, informed consent was obtained from all legally authorized representatives (LAR)/next of kin instead of the patients because of illness severity.

The study included consecutive COVID‐19 severely ill patients for the observation of ICCI and CHD in this population although the observation of high prevalence of obesity among the included patients motivated this present analysis. All patients included had confirmed COVID‐19 by real‐time reverse transcription–polymerase chain reaction positive testing and were included within the first 72 h since the initiation of invasive mechanical ventilation. Exclusion criteria included the absence of LAR consent, the absence of temporal acoustic window for TCD assessment, patients unable to undergo ICC monitoring due to lesions and/or skin infections in the sensor application region and patients with head circumference smaller than 47 cm. The study protocol was according to the Standards for Reporting of Diagnostic Accuracy Studies (STARD) statement (Supplemental Table).

Eligible subjects were identified by the ICU teams (SYW, SF, BT, EB and LMSM). As patients were included consecutively, inclusion of patients with and without obesity was spontaneous. Two assessments of CVH and ICC were performed: the first during the first 3 days from intubation and the second up to 72 h after extubation or tracheotomy without administration of sedatives; for patients who died while intubated only the first evaluation was considered. Clinical parameters, such as systemic arterial pressure, fluid balance, use of sedatives, PaO_2_ and PaCO_2_, hemoglobin and body temperature, were concomitantly recorded. One operator, without knowledge of the individual clinical features, performed all evaluations. Data on demographic characteristics, Simplified Acute Physiologic Score (SAPS) 3, use of intravenous sedatives, vasopressors and other physiological and laboratory data were also collected.

### 2.2 Intracranial compliance monitoring technique

ICC was evaluated non‐invasively by assessing cranial deformation using a specific device (B4C; Brain4care Corp., São Carlos). The B4C sensor consists of a support for a sensor bar that detects local cranial bone deformations using specific sensors. The detection of these deformations is obtained by a cantilever bar modeled through finite element calculations. Voltage meters are attached to this bar for deformation detection. Non‐invasive contact with the skull is obtained by adequate pressure directly into the scalp by means of a pin. The system is positioned in the frontotemporal region, around 3 centimeters over the first third of the orbitomeatal line; consequently, avoiding temporal superficial artery main branches and temporal muscle, providing contact of the sensor with an area of thin skin and skull, whereas slight pressure is applied to the adjustable band until optimal signal is detected.

**FIGURE 1:**
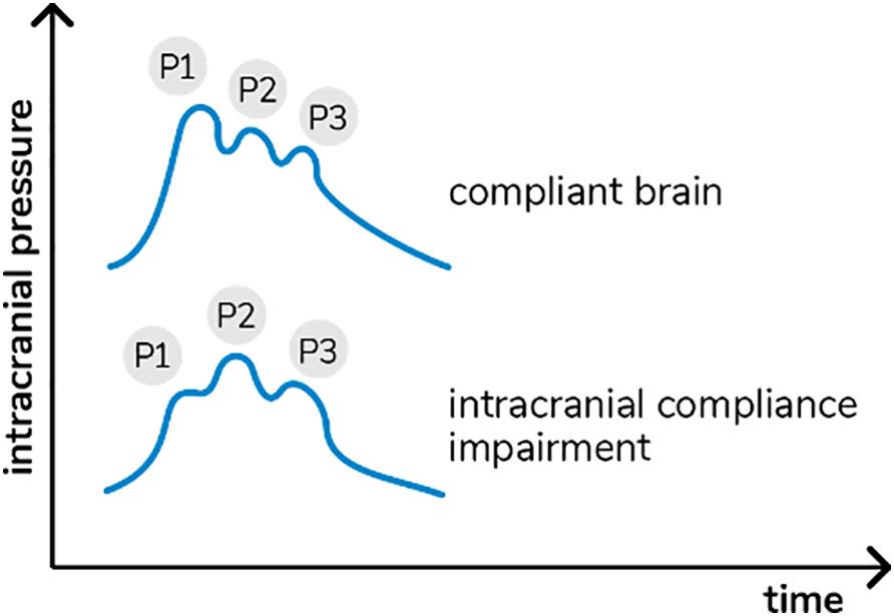
Intracranial pressure waves morphology in accordance with cerebral compliance

Variations in ICP cause deformations in the cranial bone, which are detected by the sensor bar. The device filters, amplifies and scans the sensor signal and sends the data to a mobile device. The method is completely non‐invasive and painless. In addition, it does not interfere with any routine monitoring. The waveform obtained is equivalent to ICP waveform obtained using invasive techniques, such intraparenchymal probes or external ventricular derivation,^19^ and the relation between its different components provides information on ICC.^20^ In particular, each cardiac beat corresponds to an ICP waveform composed of three peaks: arterial pulsation (P1); cerebral venous flow, which is secondary to cyclic fluctuations of arterial blood volume, reflecting intracranial compliance (P2); the aortic valve closure (P3; Figure 1).^21^

The B4C analytics system verified all data collected by the sensor, that is, ICP pulse waves morphology parameters such as the P2/P1 ratio. For this study, all calculations were performed using the mean pulse of the ICP, calculated by identifying and extracting all ICP pulses, excluding possible artifacts. The mean pulse was used to calculate the amplitudes of the P1 and P2 peaks, which were obtained by detecting the highest point of these peaks and subtracting the base value of the ICP pulse. The P2/P1 ratio was calculated by dividing the amplitude of these two points. In case of P2 > P1, ICC was defined as “abnormal”.

### 2.3 Cerebrovascular hemodynamics assessment

Conventional transcranial Doppler [(TCD) EZ‐DOP, DWL Compumetrics, Singen, Germany] was used to assess CHD.^22^ A complete evaluation of right and left cerebral hemispheres and the brainstem arteries was performed prior to the study to discard focal stenosis, using Doppler colored technique with low frequency probe (2MHz) and scanning every 1 mm of arterial extension, through the temporal, orbital, suboccipital, retro‐mastoid and submandibular windows. Hemodynamic parameters of interest were mean flow velocities in the middle cerebral arteries (mCBFV), peak systolic and final diastolic velocities, because the MCA supplies approximately 80% of cerebral blood flow. Abnormal mCBFV was identified by values < 40 or ≥ 100 cm/sec.

Using TCD, elevation of ICP was suspected when pulsatility index (PI) ≥ 1.2 (i.e., “abnormal” PI).^23^ ^,^ ^25^ PI was calculated by the following formula: PI = Sv‐Dv/Mv (Sv: systolic velocity, Dv: diastolic velocity and Mv: mean flow velocity). Moreover, TCD allows calculation of estimated CPP (eCPP) and ICP (eICP),^24^ which are significantly correlated with invasive ICP measurements.^22^, ^25^ Abnormal eICP was considered if > 20 mmHg; abnormal eCPP if ≤ 45 or ≥ 75 mmHg.

### 2.4 Outcomes

As a wide range of variables are involved in the prognosis of COVID‐19,^26^, ^27^, ^28^, ^29^ our analyses were limited to the prevalence and predictive values of ICC and CVH disturbances on early unfavorable outcome (UO); between patients included consecutively and distributed in two groups according to presence or absence of obesity. UO was a composite endpoint including either absence of weaning from mechanical ventilation (MV) or death on day 7 after inclusion in the study.

CHD and ICC impairment were identified using the different combination of TCD and B4C values; in particular, P2/P1 ratio, mCBFV, eICP, PI and eCPP were categorized and an arbitrary score was developed to describe different degrees of these alterations (Table 1). For each variable, severity was defined by a CHD/ICCI score from 1 to 4. As such, the sum of the severity score for each variable gave a score ranging from a minimum of 5 to a maximum of 20. The score was then classified as: “normal”, that is, five points, which suggested no abnormalities; “mild CHD/ICCI abnormalities”, that is, Six to seven points, which was associated with minor disturbances in one or two variables; “moderate CVH/ICC abnormalities”, that is, Eight to nine points; and “severe CHD/ICCI”, that is, ≥10 points.

**TABLE 1:** Thresholds for P2/P1 ratio, mCBFV, PI, eICP and eCPP. Progressive points were in accordance with the worst results

Points	P2/P1	mCBFV	PI	eICP	eICP	Score (sum of Each)
1	≤1	40 to 70	<1.2	<15	50 to 75	5 no CVH/ICCI
2	1.01 to 1.19	71 to 99	≥1.2	15‐20	≥75	6‐7 mild CVH/ICCI
3	≥1.2	≥100	≥1.3	21‐25	≤50	8‐9 moderate CVH/ICCI
4	≥1.4	<40	≥1.4	>25	<40	≥10 severe CVH/ICCI

Abbreviations: CVH/ICCI, cerebrovascular hemodynamics and intracranial compliance impairment; eCPP, estimated cerebral perfusion pressure; eICP, estimated intracranial pressure; PI, pulsatility index.

### 2.5 Sample size

A pilot study was performed. Using the upper confidence interval for the population variance approach to the sample size calculation, a pilot sample size between 20 and 40 was chosen, corresponding to standardized effect sizes of 0.4 and 0.7 (for 90% power based on a standard sample size calculation).^30^ Thus, considering the risk of early deaths and lack of second TCD and B4C assessment, 50 patients were enrolled to test our hypothesis.

### 2.6 Statistical analysis

The 50 patients included were separated in two groups according whether the BMI was over or under 30 kg/m^2^. Descriptive statistics were computed for all study variables. Categorical variables are presented as count (%), while continuous variables are presented as mean (± standard deviation) or median (25th–75th percentiles), according to their distribution, which was assessed through skewness and kurtosis values, as well as graphical methods. Differences between groups were assessed using a χ‐square or Fisher's exact test for categorical variables, t‐Student test for normally distributed continuous variables and Mann‐Whitney tests for asymmetrically distributed continuous variables.

Multivariable adjustment with multiple logistic regression was used to verify the independent association between obesity and ICCI with results expressed in odds ratios and their respective 95% Confidence Intervals (CI). The model was specified a priori to adjust for disease respiratory severity and overall severity. The former model included age and PaO2/FiO2 ratio and the latter included age, PaO2/FiO2 ratio, ICU admission SOFA score laboratory parameters (creatinine, bilirubin and platelets) and d‐dimer. Except for age, all other covariates were log transformed to ensure the normality of the residuals. Multiple imputation was used to handle missing data.

Final *p* values under 0.05 were considered statistically significant. All analyses were performed using the software Statistical Package for Social Sciences (IBM SPSS Statistics for Windows, version 24.0. Armonk, NY: IBM Corp.). This clinical trial (CT) study protocol was approved by the local Ethics Committee, in 19 April 2020 and registered under number NCT04429477 (available at clinicaltrials.gov).

## 3 RESULTS

### 3.1 Sample features

Overall COVID‐19 admissions between May and June were 2813, whereas ICU admissions in this period were 1579 in our institution, with 552 (34.9%) deaths (institution reference for moderate‐severe cases). Among eligible subjects, drop‐outs were 1 because of LAR refusal, and 23 because of transference from another institution over the inclusion period. TCD evaluations on CVH were performed for the entire sample, with no absence of temporal acoustic windows found.

Overall group's features are described in Table 2. 30 (60%) patients died during hospitalization, among these, 21 (42%) died in the first 4 to 29 (13 days average) days of ICU therapy, still under ventilatory support being no TCDand B4C reassessed. There were no statistical differences between survivor and non‐survivor groups, except for higher age and lower PaO2/FiO2 among deceased subjects. 29 patients reached reassessment because of MVW or tracheostomy with sedation interruption. Average length of hospitalization was 51 (4–67) days for deceased and 30 (9–70) for survivors. Seven needed tracheostomy and eight underwent re‐intubation. Respiratory rate was over 16 bpm and oxygen saturation under 94% for 48 and 31 patients, respectively. 28 days posterior to RSW, only 8 (40% of survivors) patients reached hospital discharge.

**TABLE 2:** Sample characteristics according to obesity status

Variable	General (50)	Obesity		p value
		No (27)	Yes (23)	
Age (mean ± SD)	55.9 ± 16.6	58.4 ± 18.3	53 ± 14.1	0.253
Female	22 (44)	11 (40.7)	11 (47.8)	0.615
Unfavorable outcome	33 (66)	12 (44.4)	16 (69.6)	0.075
Altered parameter				
P2/P1 ratio	31 (62)	13 (48.1)	18 (78.3)	0.029
mCBFV	33 (66)	18 (66.7)	15 (65.2)	0.914
PI	33 (66)	17 (63)	16 (69.6)	0.623
eICP	18 (36)	3 (33.3)	9 (39.1)	0.670
eCPP	28 (56)	17 (63)	11 (47.8)	0.283
Chronic kidney injury	9 (18)	3 (11.1)	6 (26.1)	0.270
Smoking	16 (32)	7 (25.9)	9 (39.1)	0.318
Cardiovascular disease	10 (20)	5 (18.5)	5 (21.7)	>0.999
Diabetes	17 (34)	7 (25.9)	10 (43.5)	0.192
Hypertension	28 (56)	13 (48.1)	15 (65.2)	0.226
Cancer	7 (14)	5 (18.5)	2 (8.7)	0.430
PaO2/2FiO2 ratio	142 (125 – 182)	149 (133 – 182)	137 (97 – 201)	0.384
Admission laboratory parameters (median and quartiles)				
Creatinine	1.1 (0.7 – 2.6)	1.1 (0.7 – 2.3)	0.9 (0.7 – 2.8)	0.946
Bilirubin	0.4 (0.2 – 0.5)	0.4 (0.3 – 0.5)	0.3 (0.2 – 0.6)	0.633
Platelets	226 (150 – 320)	227 (148 – 348)	216 (163 – 313)	0.820
D‐dimer	2622 (1250 – 6058)	3219 (1166 – 10,234)	2390 (1416 – 5629)	0.763
Length of stay (median and quartiles)	23 (13 – 30)	25 (12 – 30)	20 (14 – 30)	0.977

Abbreviations: eCPP, estimated cerebral perfusion pressure; eICP, estimated intracranial pressure; mCBFV, middle cerebral artery highest mean velocity; PI, pulsatility index; SD, standard deviation.

Twenty‐three patients were obese (Table 2). There was no difference between obese and non‐obese patients regarding age, gender, comorbidities, PaO2/2FiO2 ratio and ICU admission laboratory parameters (creatinine, bilirubin, platelets and d‐dimer). ICCI was more frequent in obese patients (78.3 vs. 48.1%, p = 0.029), although the TCD parameters (mCBFV, PI, eICP, eCPP) were similar between groups. Obese patients tended to present more unfavorable outcomes (69.6 vs. 44,4%, p = 0.075).

Besides being more present among obese patients, in the univariate analysis, ICCI was associated with admission creatinine and bilirubin as well as mCBFV, PI and eICP (Table 3). Unfavorable outcomes occurred more frequently among those with ICCI (74.2 vs. 26.3%, p = 0.001).

**TABLE 3:** Sample characteristics according to intracranial compliance status

Variable	Intracranial Compliance Impairment		p value
	No (19)	Yes (31)	
Age (mean ± SD)	51.4 ± 19.6	58.6 ± 14.1	0.137
Female	6 (31.6)	16 (51.6)	0.166
Obesity	5 (26.3)	18 (58.1)	0.029
Unfavorable outcome	5 (26.3)	23 (74.2)	0.001
Altered parameter			
mCBFV	5 (26.3)	28 (90.3)	<0.001
PI	5 (26.3)	28 (90.3)	<0.001
eICP	4 (21.1)	14 (45.2)	0.085
eCPP	10 (52.6)	18 (58.1)	0.707
Chronic kidney injury	3 (15.8)	6 (19.4)	>0.999
Smoking	4 (21.1)	12 (38.7)	0.194
Cardiovascular disease	3 (15.8)	7 (22.6)	0.722
Diabetes	7 (36.8)	10 (33.2)	0.740
Hypertension	8 (42.1)	20 (64.5)	0.121
Cancer	2 (10.5)	5 (16.1)	0.695
PaO2/FiO2 ratio	145 (125 – 216)	142 (115 – 173)	0.394
Admission laboratory parameters (median and quartiles)			
Creatinine	1.3 (0.8 – 3.0)	0.9 (0.7 – 1.7)	0.089
Bilirubin	0.6 (0.4 – 1.3)	0.3 (0.2 – 0.4)	0.006
Platelets	169 (120 – 315)	249 (178 – 329)	0.136
D‐dimer	2338 (779 – 5470)	3219 (1457 – 7343)	0.250
Length of stay (median and quartiles)	26 (12 – 37)	20 (14 – 30)	0.555

Abbreviations: eCPP, estimated cerebral perfusion pressure; eICP, estimated intracranial pressure; mCBFV, middle cerebral artery highest mean velocity; SD, standard deviation.

**FIGURE 2:**
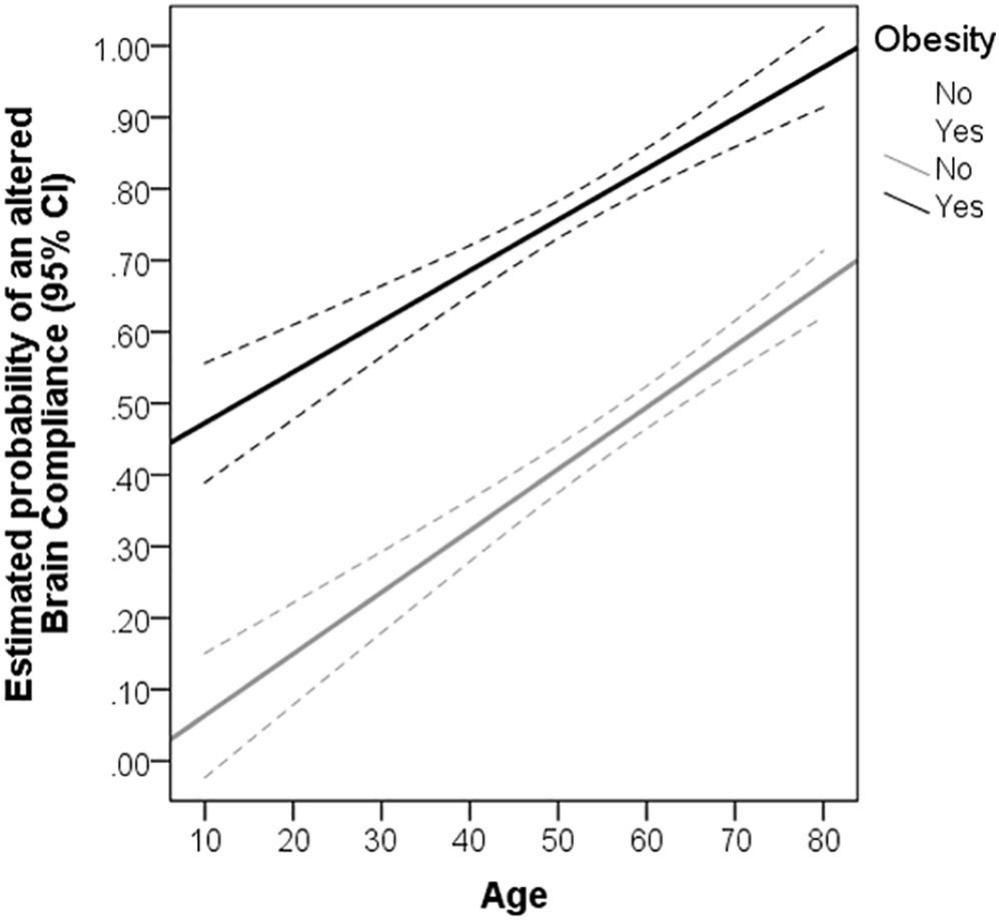
Estimated probability of intracranial compliance impairment according to obesity status and age (adjusted for PaO2/FiO2 and laboratory parameters after multiple imputation)

In the multivariable analysis, obesity maintained independent association with ICCI after adjustment for respiratory disease severity (Table 4, model 1, OR 5.47, 95% CI 1.35 ‐ 22.18, p = 0.017) and overall severity (Table 4, model 2, OR 12.35, 95% CI 1.57–97.36, p = 0.017). Concerning early outcomes, ICCI and admission d‐dimer were associated with unsuccessful MVW/death (Table 5). Moreover, older patients tended to have a higher risk of ICCI. Figure 2 depicts the estimated probability of an altered intracranial compliance according to obesity status and age after multivariable adjustment.

**TABLE 4:** Multivariable analysis for the predictors of intracranial compliance impairment

Variable	Coef.	SE	Wald	OR	95% CI	*p* value
**Model 1**						
Age (per year)	0.04	0.02	2.86	1.04	0.99 – 1.09	0.091
Obesity	1.70	0.71	5.66	5.47	1.35 ‐ 22.18	0.017
PaO2/FiO2 (Log)	−1.41	2.48	0.32	0.25	0.01 – 31.59	0.571
**Model 2**						
Age (per year)	0.066	0.037	‐	1.07	0.99 – 1.15	0.076
Obesity	2.514	1.049	‐	12.35	1.57 – 97.36	0.017
PaO2/FiO2 (Log)	‐0.269	2.934	‐	0.76	0.01 – 246.83	0.927
Admission lab (Log)						
Creatinine	−2.200	2.533	‐	0.11	0.01 – 19.96	0.393
Bilirubin	0.047	1.703	‐	1.05	0.03 – 35.22	0.978
Platelets	3.493	2.465	‐	32.90	0.25 – 4375.69	0.160
D‐dimer	1.250	0.831	‐	3.492	0.68 – 17.87	0.133

Abbreviations: CI, confidence interval; Coef., coefficient; SE, standard error; OR, odds ratio.

**TABLE 5:** Multivariable analysis for the predictors of unfavorable outcome

Variable	Coef.	SE	OR	95% CI	*p* value
Age (per year)	0.05	0.03	1.06	1.00 – 1,12	0.070
Obesity	1.49	0.93	4.44	0.71 – 27,55	0.110
ICCI	2.02	0.89	7.52	1.31 – 43,12	0.024
Admission d‐dimer (Log)	1.75	0.88	5.74	1.02 – 32,29	0.047
SOFA score	0.06	0.25	1.06	0.65 – 1,72	0.816

Abbreviations: Coef., Coefficient; SE, Standard error; OR, Odds ratio; CI, Confidence interval; ICCI, intracranial compliance impairment.

## 4 DISCUSSION

In the present study, ICCI was significantly more present among patients with obesity. CHD prevalence was not different between groups, probably because our entire sample was composed by SARS patients, included in early stages of respiratory function depression and mechanical ventilation support, when CHD is commonly observed.^31^ Survival and mechanical ventilatory support successful weaning were also significantly higher among non‐obese subjects.

Despite of our exploratory study design, prevalence of obesity (46%) in our sample was considerably higher than Brazilian overall population obesity rate (20.7%),^32^ but similar to previous studies reported, which have noticed increased risk of hospitalization, severe disease and invasive mechanical ventilation in COVID‐19.^18^, ^33^, ^34^ Therefore, obesity was pointed as a clinical predictor for adverse outcomes.^33^, ^34^ Physicians must be alert to these early indicators to identify critical patients.^18^, ^35^, ^36^

In our cohort, both groups (lean and obese) were similar in disease severity, whereas ICCI was sensibly more present among obese than lean patients. CHD given by altered mCBFV and PI, as a reflex of compromised cerebral perfusion pressure was also correlated with ICCI. Therefore, it seems to be a chain between obesity and disturbances in intracranial compliance and cerebral circulation. Although unfavorable outcomes were more frequently seen in patients with obesity, the difference did not reach statistical significance (*p* = 0.07). The small sample size may have left the study unpowered to detect statistical significance.

The correlation between obesity and ICH is sheltered on the considerable prevalence of obesity among *pseudotumor cerebri* (IIH) patients. Moreover, for non‐obese subjects, a gaining of 5–15% of body weight^37^ or pregnancy^38^ have been recognized as risk factors for IIH. These patients frequently complain of chronic headaches and have papilledema and high opening pressure at lumbar puncture, but not in all cases.^6^ In fact, this syndrome presentation may vary widely, with possibility of late visual impairment in previously asymptomatic subjects.^8^ Weight loss is the only established disease modifying therapy in IIH. A systematic review of the IIH cases treated with bariatric surgery report 100% resolution of papilledema and 90% experience headache improvement.^15^, ^39^

Notwithstanding chronic ICH to the date cannot be proven as part of the mechanisms for CNS degeneration in obesity, the present study brings to light this hypothesis. Current evidence indicate metabolic‐inflammatory syndrome^40^ with mitochondrial dysfunction and oxidative stress to degenerate not only the brain, but even the spinal cord^41^ of obese patients. This has a significant clinical implication as obesity being a strong risk factor for brain atrophy and neurodegenerative diseases.^42^, ^43^ A recent study observed that the ratio of thigh muscle to visceral fat was positively correlated with the volumes of entorhinal cortex, temporal pole, and inferior temporal gyrus related to cognition. Additionally, the ratio of thigh muscle to visceral fat was positively correlated with the volumes of cerebellum and pallidum related to movement.^44^

With reference to COVID‐19 and the brain, plenty of findings have been described.^45^, ^46^, ^47^ Central nervous thrombosis seems to have an increased relative risk.^45^ Likewise, large vessel occlusion of ischemic stroke patients, intracerebral hemorrhage in locations not associated with systemic chronic hypertension, as callosal and/or juxtacortical were all frequent among patients with neurological clinical manifestations. Features of hemorrhagic necrotizing encephalitis were detected in 28.8% of patients with meningoencephalitis.^46^ COVID‐19 also may have the potential to incite or accelerate neurodegeneration.^47^ Patients with previous multiple sclerosis, Parkinson's or Alzheimer's diseases were significantly present amid hospitalized COVID‐19. Although mortality was higher only for DA patients (47), worsening of all these diseases after SARS‐COV‐2 infection have been observed.^48^

Considering obesity and COVID‐19, justifications for higher aggressive disease in this population rely on cardiovascular, hematologic, autonomic, endocrine, metabolic, immune and inflammatory mechanisms.^49^ The amount of adipose tissue, rich in angiotensin‐converting enzyme 2 (ACE2) receptors, the association between obesity and with type 2 diabetes which also leads to an increase in ACE2 receptors and an overactivation of the renin‐angiotensin‐aldosterone system in adipose tissue result in difficult systemic hemodynamic control.^49^ COVID‐19 is clearly linked with coagulopathy, and in case of obesity higher risk of venous thromboembolism.^49^ In obesity there is reduced macrophage activation, increased proinflammatory cytokine production, and impaired B and T cell activation.^50^

The circuit obesity‐ICH‐COVID‐19 may find its vertex in invasive mechanical ventilation. Ventilating obese patients is quite challenging due to decreased diaphragmatic excursion, decreased expiratory reserve volume, and decreased lung functional capacity.^50^ Besides proper arterial pressure management, ventilation has a crucial role on the prevention of brain's secondary damages even when primary disease is not on the CNS, as sepsis per example,^51^ however, adjustment of these parameters are not enough if neurovascular coupling has been lost.^52^ The limitation of chest wall and respiratory system elastances are correlated to elevation in ICP when positive‐end‐expiratory‐pressure is applied,^53^ what is expected for our population with obesity and SARS. These observations may justify the higher prevalence of ICCI among obese subjects in our study.

Finally, pronation positioning, what was often applied for ventilation of COVID‐19 severe patients,^54^ normally takes patients off the ideal 30° of head elevation, for ideal CSF transit and venous return.^55^ Perhaps the ideal positioning during pronation would be reverse Trendelenburg, although this was not verified in this study.

This study had several limitations to be acknowledged. Our findings showed association and not causality between UO and alterations in cerebral hemodynamics. Routine daily assessment of these variables as potential therapies to restore “normal” brain hemodynamics will be in this setting. Second, the correlation between the B4C system and other surrogate of brain compliance, such as invasive ICP monitoring, has not been validated yet. The thresholds applied in our study were, however, extrapolated from the previous knowledge on ICC and ICP research. As this system acquires intracranial information through an extracranial technique, precaution is needed. Third, we could not perform neurological imaging during the study period, which is a hindrance to elucidate the etiology of the alterations in intracranial pressure pulse waveform in COVID‐19, whether as primary CNS injury or secondary to respiratory or other systemic complications. Forth, availability of operators to assess brain hemodynamics restricted the study exclusively for severe COVID‐19 admitted to the ICU. Finally, we used a composite endpoint of early systemic dysfunction, which is not specific for brain damage; long‐term neurological assessment should be evaluated in future studies in association with early disturbances of brain perfusion and compliance.

## 5 CONCLUSION

In the present study, intracranial compliance impairment was significantly more observed among obese subjects and may have contributed for SARS COVID‐19 worsen prognosis. Further studies with wider samples are needed to determine ICCI prevalence in this population precisely.

## CONFLICT OF INTEREST

There is no conflict of interest that could be perceived as prejudicing the impartiality of the research reported.

## FUNDING INFORMATION

This research did not receive any specific grant from any funding agency in the public, commercial or not‐for‐profit sector.

## AUTHOR CONTRIBUTION

Sérgio Brasil conceived and collected data of the study. Sérgio Brasil and Alessandra Covallero Renck wrote the manuscript. Davi Jorge Fontoura Solla statistical analysis. Sâmia Yasin Wayhs, Sérgio Fonseca, Estevão Bassi, Bruno Lucena and Bruno Martins Tomazini collected digital data. Elaine Maria Frade Costa, Manoel Jacobsen Teixeira, Ricardo De Carvalho Nogueira, Wellingson Paiva and Luiz Marcelo Sá Malbouisson revised the manuscript.
